# Matrix metalloproteinase activation and TNF upregulation characterize the sclerotic phase of aortic valve disease

**DOI:** 10.3389/bjbs.2026.16540

**Published:** 2026-05-13

**Authors:** Elizabeth Chan-Delgado, Claudia Lerma, Juan C. Echeverría, Andrea Toledo, José M. Torres-Arellano, Oscar Infante, Rafael Bojalil, Jorge E. Cossío-Aranda, Nydia Ávila-Vanzzini, Luis M. Amezcua-Guerra, Rashidi Springall

**Affiliations:** 1 Master’s Program in Chemical-Biological Sciences, National School of Biological Sciences, National Polytechnic Institute, Mexico City, Mexico; 2 Department of Immunology, National Institute of Cardiology Ignacio Chávez, Mexico City, Mexico; 3 Red MEDICI, Faculty of Higher Studies Iztacala, National Autonomous University of Mexico, Mexico City, Mexico; 4 Department of Molecular Biology, National Institute of Cardiology Ignacio Chávez, Mexico City, Mexico; 5 Department of Electrical Engineering, Metropolitan Autonomous University-Iztapalapa, Mexico City, Mexico; 6 Department of Electromechanical Instrumentation, National Institute of Cardiology Ignacio Chávez, Mexico City, Mexico; 7 Health Care Department, Metropolitan Autonomous University-Xochimilco, Mexico City, Mexico; 8 Cardiology Outpatient Clinic, National Institute of Cardiology Ignacio Chávez, Mexico City, Mexico; 9 Medimanage Research, Mexico City, Mexico; 10 School of Medicine, La Salle University, Mexico City, Mexico

**Keywords:** aortic valve sclerosis, aortic valve stenosis, extracellular matrix, metalloproteinases, tumor necrosis factor

## Abstract

**Introduction:**

Aortic valve sclerosis (AVSc) is an active pathological process driven by extracellular matrix remodeling, consistent with a potentially reversible early stage of aortic valve disease.

**Methods:**

In this cross-sectional study of 168 participants (29, normal aortic valve [NAV]; 98, AVSc; 41, aortic stenosis [AS]), serum levels of matrix metalloproteinase (MMP)-1, -2, -3, and -9, tissue inhibitor of metalloproteinases-1 (TIMP-1), tumor necrosis factor (TNF), interleukin-6 (IL-6), and transforming growth factor-beta (TGF-β) were measured. Multivariable adjustments were performed.

**Results:**

Compared with AS, AVSc was associated with higher circulating MMP-9 levels, with MMP-9 also increased relative to NAV. TIMP-1 concentrations were reduced in AVSc compared with both AS and NAV. TNF levels were higher in AVSc than in AS, while IL-6 and TGF-β did not differ among groups. Notably, MMP-9/TIMP-1 ratio was markedly increased in AVSc.

**Discussion:**

Our findings suggest that AVSc may exhibit an active proteolytic and inflammatory profile amenable to targeted anti-inflammatory and anti-proteolytic interventions.

## Introduction

Calcified aortic valve disease (CAVD) encompasses a continuum of pathological changes ranging from aortic valve sclerosis (AVSc), the early and subclinical stage, to aortic valve stenosis (AS), the advanced hemodynamically significant phase. The prevalence of CAVD increases markedly with age, from approximately 0.2% in individuals aged 50–59 years to nearly 10% among those aged 80–89 years for AS, and from 25% to 40% between ages 65 and 75 for AVSc [1]. Once regarded as a passive, degenerative process of aging, CAVD is now recognized as an active, tightly regulated disease driven by valvular interstitial cells residing in the fibrosa layer of the aortic valve [2].

During the AVSc phase, fibrocalcific remodeling occurs without impairing valve function, and hemodynamic and echocardiographic parameters typically remain within normal limits. Over a prolonged subclinical period, however, progressive thickening and focal calcification can culminate in AS, characterized by increased transvalvular gradients and a reduced aortic valve area [3]. Because AVSc is often asymptomatic and underdiagnosed, most patients are identified only once significant stenosis of the aortic valve has developed, when surgical or interventional valve replacement remains the sole effective therapy and mortality is high if it is left untreated. Consequently, elucidating the molecular and inflammatory mechanisms underlying early CAVD is crucial to identifying potential biomarkers and therapeutic targets capable of halting or delaying disease progression [4].

AVSc shares key clinical, structural, and molecular features with atherosclerosis, suggesting that both conditions may arise from overlapping pathogenetic mechanisms. In each, disease initiation is marked by lipids accumulation within the subendothelial layer–occurring in the arterial wall in atherosclerosis and in the arterial valve leaflets in AVSc. Lipids deposition promotes local inflammation, macrophage activation, and foam cell formation, ultimately contributing to calcification. In both settings, progressive mineralization increases tissue stiffness–arterial or valvular–reducing compliance and generating hemodynamic obstruction [5, 6].

In atherosclerosis, inflammation is a central driver of disease progression. Endothelial dysfunction, triggered by cardiovascular risk factors such as hypertension, diabetes mellitus, hyperlipidemia, and smoking, initiates endothelial activation and the recruitment of immune cells, including macrophages and T cells, into the intima. Once infiltrated, these cells release proinflammatory cytokines, notably tumor necrosis factor (TNF) and interleukin-6 (IL-6), which amplify local inflammation and promote the osteogenic differentiation of resident progenitor cells, thereby accelerating vascular calcification [7, 8]. In parallel, these cytokines stimulate the expression of matrix metalloproteinases (MMPs), a family of proteolytic enzymes that degrade extracellular matrix (ECM) components and contribute to maladaptive tissue remodeling [9].

The convergence of inflammatory and proteolytic pathways in both atherosclerosis and AVSc underscores the role of chronic, unresolved inflammation in pathological tissue remodeling. In AVSc, inflammation is thought to originate at the valvular endothelium, where activated endothelial cells may release cytokines that attract immune cell infiltration and enhance MMP expression, leading to ECM degradation and progressive thickening of the valve leaflets [6]. These processes facilitate the formation of early sclerotic lesions and may represent the initial step in a continuum culminating in calcific AS [10].

Based on this framework, the present study aimed to characterize the inflammatory and tissue-remodeling profile associated with AVSc by evaluating circulating mediators, including cytokines, MMPs, and the tissue inhibitor of metalloproteinases-1 (TIMP-1). Comparisons were performed with individuals presenting well-established AS and with healthy participants displaying normal aortic valve (NAV) morphology and function.

## Materials and methods

### Study design and participants

We conducted a cross-sectional study at the *Instituto Nacional de Cardiología Ignacio Chávez* (Mexico City, Mexico), including participants representing three stages of CAVD: NAV, AVSc, and AS.

Clinically healthy volunteers were recruited for the NAV and AVSc groups from hospital staff and relatives of patients, provided they had no known cardiac comorbidities. Each volunteer underwent a transthoracic echocardiogram and was classified as NAV or AVSc based on imaging results. The AS group consisted of patients with a previous diagnosis of calcified AS who were candidates for elective valve replacement and were consecutively recruited from the outpatient cardiology clinic.

A total of 168 participants were initially enrolled: 127 clinically healthy volunteers and 49 patients with AS. After clinical screening and echocardiographic evaluation, 98 volunteers were classified as AVSc (grades 1–2, characterized by focal areas of increased echogenicity and leaflet thickening without motion restriction), and 29 as NAV (absence of aortic valve abnormalities). Among the AS patients, identified as described below, 8 were excluded due to other concomitant valvular disease detected by echocardiography, yielding 41 patients in the final AS group. All procedures were performed prior to valve replacement surgery.

Clinical data, including hypertension, dyslipidemia, and smoking status, were obtained from medical records. In the case of the study volunteers, a targeted medical history was taken prior to the echocardiogram. Exclusion criteria comprised coronary artery disease, renal or hepatic dysfunction, acute infections within the previous month, influenza vaccination within the preceding 6 months, autoimmune disease (e.g., systemic lupus erythematosus, rheumatoid arthritis), and moderate-to-severe mitral or tricuspid valve disease. Patients with bicuspid aortic valves or rheumatic valve disease were also excluded.

### Echocardiographic assessment

Transthoracic echocardiography was performed by a single experienced cardiologist blinded to clinical and laboratory data, using a commercial ultrasound system (iE33; Philips Healthcare, Bothell, WA, USA). Two-dimensional imaging and Doppler modalities (pulsed-wave and continuous-wave) were used to obtain the following parameters: peak aortic valve velocity (Vmax, m/s), mean and peak transvalvular pressure gradients (mmHg), aortic valve area (AVA, cm^2^) and AVA indexed to body surface area (AVA index, cm^2^/m^2^), and left ventricular ejection fraction (LVEF, %). The aortic valve was visualized in parasternal long- and short-axis views. Peak and mean velocities and gradients were measured from apical three- and five-chamber views using pulsed and continuous-wave Doppler, as appropriate. LVEF was determined from apical four- and two-chamber images using the modified Simpson’s biplane method. AVA was calculated by the continuity equation and indexed to body surface area. All measurements adhered to current American Society of Echocardiography and the European Association of Cardiovascular Imaging recommendations to ensure reproducibility [11].

AVSc was defined as a peak aortic valve velocity <2.0 m/s, with focal areas of increased echogenicity and thickened leaflets without restriction of motion or left ventricular outflow obstruction, and with preserved forward flow. Moderate AS was defined by an AVA of 1.0–1.5 cm^2^ or a mean transvalvular gradient of 25–40 mmHg, while severe AS was defined by an AVA <1.0 cm^2^ or a mean gradient >40 mmHg under normal flow conditions [12].

### Blood sampling and biomarker quantification

Peripheral venous blood (10 mL) was collected from each participant after an overnight fast. Samples were centrifuged at 3,000 rpm for 15 min at 4 °C, and serum aliquots (500 µL) were stored at −70 °C until analysis.

Serum concentrations of MMP-1, MMP-2, MMP-3, MMP-9, TIMP-1, IL-6, TNF, and transforming growth factor-beta (TGF-β) were quantified using commercially available enzyme-linked immunosorbent assay kits (R&D Systems, Minneapolis, MN, USA), following the manufacturer’s instructions. All measurements were performed in duplicate, and intra- and inter-assay coefficients of variation were <10%.

### Statistical analysis

Data distribution was assessed using the Shapiro-Wilk test. Because most variables were non-normally distributed, continuous variables were expressed as median (interquartile range, IQR: 25th-75th percentiles) and compared among groups using the Kruskal-Wallis test followed by Dunn’s *post hoc* test. Categorical variables were expressed as absolute frequencies and percentages and compared using the chi-square test.

To control for multiple testing, *p* values were adjusted using the Benjamini-Hochberg false discovery rate (FDR) procedure, with a prespecified FDR threshold of 0.05. All reported *p* values from pairwise comparisons were included in the adjustment, and adjusted *p* values were presented as *q* values.

To assess whether the association between circulating biomarkers and disease stage was independent of potential confounders, multivariable linear regression analyses were performed. Biomarkers were analyzed as dependent variables after logarithmic transformation. Disease stage was included as the main independent variable using dummy coding, with NAV as the reference category; additional models were constructed to directly compare AVSc and AS. Models were adjusted for age, sex, body mass index, systolic blood pressure, fasting glucose, and LDL-cholesterol. Regression coefficients (β) with 95% confidence intervals (CI) were reported.

All analyses were two-tailed, with a significance level set at *p* < 0.05. Analyses and data visualization were performed using GraphPad Prism version 10.3.1 (GraphPad Inc; San Diego, CA, USA) and R software (R Foundation for Statistical Computing; Vienna, Austria).

## Results

A total of 168 participants (49.46% male) were included in the study, comprising 29 individuals with NAV, 98 with AVSc, and 41 with AS (Table 1).

**TABLE 1:** Main clinical and echocardiographic data of study participants.

Characteristic	NAV (n = 29)	AVSc (n = 98)	AS (n = 41)	*p*-value
Age, years	42 (31–45)	46 (35–51)	63 (58–66)	**< 0.001**
Male, n (%)	17 (58)	42 (42)	24 (58)	0.133
BMI, kg/m^2^	25.6 (24.6–29.2)	27.0 (24.8–30.1)	29.5 (27.1–32.9)	**0.003**
Heart rate, bpm*	63 (57–68)	62 (55–68)	64 (56–71)	0.569
SBD, mmHg*	111 (110–118)	118 (110–122)	130 (110–144)	**< 0.001**
DBP, mmHg*	78 (70–80)	80 (70–80)	80 (70–80)	0.250
Comorbidities, n (%)				
• Hypertension	1 (3)	4 (4)	21 (51)	**< 0.001**
• Diabetes mellitus	0	1 (1)	9 (21)	**< 0.001**
• Current smoking	4 (13)	23 (23)	15 (36)	0.082
Laboratory results				
• Glucose, mg/dL	88 (84–94)	89 (82–94)	97 (92–107)	**< 0.001**
• Albumin, g/dL	4.5 (4.3–4.6)	4.4 (4.3–4.6)	4.4 (4.1–4.6)	0.769
• Total cholesterol, mg/dL	185 (172–204)	193 (169–215)	175 (152–202)	0.270
• HDL-C, mg/dL	41 (35–47)	45 (39–50)	44.6 (36–50)	0.329
• LDL-C, mg/dL	120 (104–142)	126 (107–146)	103 (80–131)	**0.010**
• Triglycerides, mg/dL	138 (107–160)	128 (91–168)	137 (96–187)	0.879
• hsCRP, mg/L	2.0 (0.9–3.0)	1.8 (0.9–3.5)	1.2 (0.8–6.8)	0.964
• Hematocrit, %	46.4 (44.0–47.4)	44.5 (42.5–47.8)	43.5 (40.4–45.9)	**0.044**
• Leukocytes, 1 × 10^9^/L	6.6 (5.7–7.3)	6.3 (5.6–7.3)	6.6 (5.6–7.2)	0.554
• Neutrophils, 1 × 10^9^/L	3.8 (3.3–4.4)	3.5 (3.0–4.2)	4.0 (3.5–4.8)	**0.033**
• Platelets, 1 × 10^9^/L	235 (204–277)	246 (207–300)	199 (173–262)	**0.002**
Main therapies, n (%)				
• ACEi/ARBs	0	1 (1)	17 (41)	**< 0.001**
• Statins	0	0	13 (31)	**< 0.001**
• ASA/P2Y12i	0	9 (9)	15 (36)	**< 0.001**
Echocardiographic findings				
• AVA, cm^2^	4.2 (4.0–4.2)	4.1 (3.9–4.2)	0.7 (0.5–1.3)	**0.001**
• AVA index, cm^2^/m^2^	2.1 (2.0–2.4)	2.2 (2.1–2.4)	0.3 (0.2–0.7)	**< 0.001**
• Aortic annulus, cm^2^	2.1 (1.9–2.2)	2.0 (1.9–2.2)	2.1 (2.0–2.3)	0.782
• mPG, mmHg	3 (2–3)	3 (3–4)	38 (24–70)	**< 0.001**
• Vmax AoV, m/s	1.2 (1.1–1.3)	1.2 (1.1–1.4)	3.9 (3.1–5.3)	**< 0.001**
• LVEF, %	65 (58–67)	62 (60–68)	56 (52–60)	**< 0.001**

All data are presented as median (interquartile range, 25th–75th percentile) unless otherwise specified. Significant *p*-values are in bold.

* Measured at supine position.

Definitions: ACEi, Angiotensin-converting enzyme inhibitors; ARBs, Angiotensin II receptor blockers; ASA, Acetylsalicylic acid; AS, Aortic valve stenosis; AVA, Aortic valve area; AVSc, Aortic valve sclerosis; BMI, Body mass index; DBP, Diastolic blood pressure; HDL-C, High-density lipoprotein cholesterol; hsCRP, High-sensitivity C-reactive protein; LDL-C, Low-density lipoprotein cholesterol; LVEF, Left ventricular ejection fraction; mPG, mean transvalvular pressure gradient; NAV, normal aortic valve; P2Y12i, P2Y12 receptor inhibitors; SBP, Systolic blood pressure; Vmax AoV, Maximum transvalvular velocity in the aortic valve.

Patients with AS were significantly older than those with AVSc and NAV (median age: 63 *versus* 46 *versus* 42 years, respectively; *p* < 0.001). Body mass index was progressively higher across the groups (29.5 *versus* 27.0 *versus* 25.6 kg/m^2^; *p* = 0.003), while systolic blood pressure (130 *versus* 118 *versus* 111 mmHg; *p* < 0.001) and fasting glucose levels (97 *versus* 89 *versus* 88 mg/dL; *p* < 0.001) were also elevated in AS compared with AVSc and NAV, respectively. Conversely, LDL-cholesterol concentrations were lower in AS than in AVSc and NAV (103 *versus* 126 *versus* 120 mg/dL; *p* = 0.010). No significant differences were found in total cholesterol, HDL-cholesterol, triglycerides, or C-reactive protein levels.

Transthoracic echocardiography revealed marked differences among the groups (*p* < 0.001 for all parameters). Compared with AVSc and NAV, AS patients exhibited a markedly reduced AVA (0.7 *versus* 4.1 *versus* 4.2 cm^2^) and AVA index (0.3 *versus* 2.2 *versus* 2.1 cm^2^/m^2^), as well as lower LVEF (56% *versus* 62% *versus* 65%). Conversely, mean transvalvular pressure gradient (38 *versus* 3 *versus* 3 mmHg), and peak transvalvular velocity (3.9 *versus* 1.2 *versus* 1.2 m/s) were higher in AS.

**FIGURE 1:**
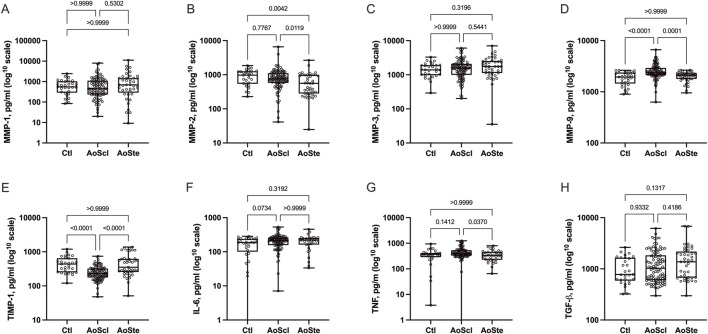
Serum levels of selected matrix metalloproteinases (MMPs) were measured in patients with aortic valve sclerosis (AVSc), aortic stenosis (AS), and in subjects with normal aortic valves (NAV). Distinct patterns were observed among groups, with MMP-9 **(D)** and MMP-2 **(B)** significantly increased, and TIMP-1 **(E)** significantly decreased in the AVSc group. Tumor necrosis factor [TNF; **(G)**] levels were also elevated in AVSc patients compared to those with AS. No differences were found in MMP-1 **(A)**, MMP-3 **(C)**, interleukin-6 [IL-6; **(F)**], and transforming growth factor-beta [TGF-β; **(H)**] levels. These findings suggest that MMP dysregulation and enhanced inflammation are early features in the pathogenesis of calcified aortic valve disease. Data are presented as median and interquartile range. Values are plotted on a log10 scale to facilitate visualization.

As shown in Figure 1, patients with AVSc had significantly higher MMP-2 concentrations than those with AS (767, 582–1096 *versus* 559, 283–983 pg/mL; *p* = 0.011) and also of MMP-9 (2,375, 2,135–2,926 *versus* 2090, 1792–2,287 pg/mL; *p* < 0.001). Compared with NAV, AVSc patients also displayed higher MMP-9 levels (2,375, 2,135–2,926 *versus* 1948, 1516–2,345 pg/mL; *p* < 0.001), while MMP-2 levels were similar (767, 582–1096 *versus* 977, 552–1244 pg/mL; *p* = 0.776). No significant differences among groups were observed for MMP-1 or MMP-3. TIMP-1 levels were significantly lower in AVSc (233, 178–319 pg/mL) than in AS (354, 257–613 pg/mL; *p* < 0.001) and NAV (436, 254–643 pg/mL; *p* < 0.001). Among cytokines, TNF levels were higher in AVSc than in AS (403, 329–526 *versus* 328, 223–450 pg/mL; *p* = 0.037), whereas IL-6 and TGF-β levels did not differ significantly among groups.

**FIGURE 2:**
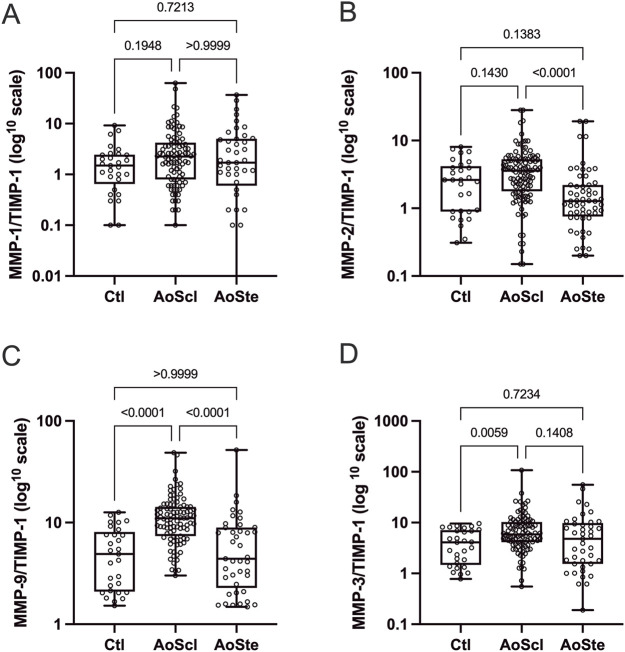
Ratios between serum concentrations of matrix metalloproteinases (MMPs) and their endogenous inhibitor (TIMP-1) were calculated to assess the proteolytic balance among study groups. Patients with aortic valve sclerosis (AVSc) displayed a distinctive MMP/TIMP-1 profile–particularly MMP-9/TIMP-1 **(D)**, and to a lesser extent MMP-2/TIMP-1 **(B)** and MMP-3/TIMP-1 **(C)**–characterized by elevated proteolytic potential compared with patients with aortic stenosis (AS) and subjects with normal aortic valves (NAV). No differences were observed in MMP-1/TIMP-1 ratio **(A)**. This pattern is consistent with active extracellular matrix remodeling during the early, inflammatory stage of calcified aortic valve disease. Data are presented as median and interquartile range. Values are plotted on a log10 scale to facilitate visualization.

As shown in Figure 2, AVSc patients exhibited markedly increased MMP-2/TIMP-1 (3.5, 1.9–5.1) and MMP-9/TIMP-1 ratios (11.0, 7.4–14.2) compared with AS (MMP-2/TIMP-1: 1.1, 0.7–2.0; *p* < 0.001; MMP-9/TIMP-1: 4.4, 2.4–8.8; *p* < 0.001). Relative to NAV (4.8, 2.1–7.6), AVSc patients also showed higher MMP-9/TIMP-1 ratios (*p* < 0.001). A higher MMP-3/TIMP-1 ratio was observed in AVSc compared with NAV (5.8, 4.1–10.2 *versus* 4.0, 1.5–6.8; *p* = 0.005). No other significant differences in proteolytic ratios were detected among the study groups.

After adjustment for multiple comparisons using the Benjamini-Hochberg FDR method, the main findings remained statistically significant. Comparisons showed that MMP-9 levels (AVSc *versus* AS and AVSc *versus* NAV), TIMP-1 levels (AVSc *versus* AS and AVSc *versus* NAV), and the MMP-2/TIMP-1 and MMP-9/TIMP-1 ratios (AVSc *versus* AS and AVSc *versus* NAV) retained high statistical significance (*q* values ∼0.001 for all). Likewise, the MMP-3/TIMP-1 ratio in AVSc compared with NAV remained significant after adjustment (*q* = 0.006), as did MMP-2 levels in AVSc compared with AS (*q* = 0.013). TNF levels in AVSc compared to AS also remained significant, although with a marginal value (*q* = 0.040). In contrast, MMP-2 levels between AVSc and NAV remained non-significant after adjustment (*q* = 0.776).

In multivariable models using NAV as the reference category (Table 2), AVSc remained independently associated with a higher MMP-9/TIMP-1 ratio (β = 0.78; 95% CI, 0.52 to 1.04; *p* < 0.001), higher MMP-9 levels (β = 0.27; 95% CI, 0.12 to 0.43; *p* < 0.001), and lower TIMP-1 concentrations (β = −0.49; 95% CI, −0.73 to −0.24; *p* < 0.001). In contrast, MMP-2 levels and the MMP-2/TIMP-1 ratio were not independently associated with AVSc after adjustment. Additional models directly comparing AVSc and AS showed that AVSc remained associated with a higher MMP-9/TIMP-1 ratio and lower TIMP-1 levels. When compared with NAV, AS exhibited a different biomarker profile, with no significant increase in MMP-9/TIMP-1 ratio.

**TABLE 2:** Multivariable linear regression analysis of circulating biomarkers according to disease stage.

Dependent variable	Comparison	β coefficient (95% CI)	*p*-value
Log (MMP-9/TIMP-1)	AVSc vs. NAV	0.78 (0.52–1.04)	**< 0.001**
​	AVSc vs. AS	0.59 (0.24–0.93)	**< 0.001**
​	AS vs. NAV	0.00 (−0.24 to 0.00)	0.110
Log (MMP-9)	AVSc vs. NAV	0.27 (0.12–0.43)	**< 0.001**
​	AVSc vs. AS	0.15 (−0.00–0.30)	0.057
​	AS vs. NAV	0.00 (−0.13 to 0.00)	**< 0.001**
Log (TIMP-1)	AVSc vs. NAV	−0.49 (−0.73 to −0.24)	**< 0.001**
​	AVSc vs. AS	−0.44 (−0.73 to −0.15)	**0.003**
​	AS vs. NAV	0.00 (−0.12 to 0.00)	**< 0.001**
Log (MMP-2/TIMP-1)	AVSc vs. NAV	0.34 (−0.13–0.72)	0.140
​	AVSc vs. AS	0.60 (0.11–1.09)	**0.017**
​	AS vs. NAV	0.00 (−0.28 to 0.00)	**0.017**
Log (MMP-2)	AVSc vs. NAV	0.07 (−0.03–0.16)	0.318
​	AVSc vs. AS	0.16 (−0.24–0.56)	0.432
​	AS vs. NAV	0.00 (−0.08 to 0.00)	**< 0.001**

Models adjusted for age, sex, body mass index, systolic blood pressure, fasting glucose, and LDL-cholesterol. Significant *p*-values are in bold.

Definitions: AS, Aortic valve stenosis; AVSc, Aortic valve sclerosis; CI, confidence interval; MMP, matrix metalloproteinase; NAV, normal aortic valve; TIMP, tissue inhibitor of metalloproteinase.

## Discussion

This study supports the concept that CAVD progresses through distinct yet pathophysiologically connected stages, with AVSc representing an early, biologically active phase that precedes hemodynamically significant stenosis. Our findings indicate that the proteolytic imbalance observed in AVSc persists after adjustment for major clinical and metabolic confounders, as AVSc remained independently associated with higher MMP-9 levels, lower TIMP-1 concentrations, and, notably, an increased MMP-9/TIMP-1 ratio. These results are consistent with a shift toward enhanced ECM turnover in early disease stages. In contrast, the lack of independent association for MMP-2 and IL-6 may indicate a more limited or context-dependent contribution of these biomarkers after accounting for confounding factors. The attenuated or non-significant associations observed in established AS further support the interpretation that AVSc represents a biologically active phase characterized by inflammation-driven extracellular matrix remodeling, rather than a mere precursor state. Overall, these findings suggest that the imbalance between proteolytic enzymes and their inhibitors is not fully explained by traditional cardiovascular risk factors and may reflect intrinsic disease mechanisms operative during the early stages of valvular degeneration.

At the molecular level, our results demonstrate a selective inflammatory activation in AVSc, characterized by significantly elevated circulating TNF compared with AS, whereas IL-6 and TGF-β remained unchanged. This cytokine pattern underscores TNF as an upstream regulator of valvular inflammation, capable of activating endothelial cells, inducing adhesion molecule expression, and promoting LDL uptake. Collectively, these effects facilitate immune cell recruitment and early lesion development [13]. By analogy with atherosclerosis, enhanced TNF signaling in valvular endothelium may promote leukocyte infiltration and early matrix remodeling, reinforcing that AVSc represents an immune-mediated and metabolically driven stage of valve disease rather than a passive, age-dependent degenerative process [14].

Beyond inflammation, our data reveal a profound shift in the proteolytic balance in AVSc. Elevated circulating MMP-9 levels, together with reduced TIMP-1, yielded markedly increased MMP-9/TIMP-1 ratio, indicating a net proteolytic state favoring ECM degradation. This contrasts with the fibrotic, calcified phenotype of advanced AS and agrees with previous reports showing enhanced MMP expression and oxidized LDL accumulation in sclerotic valves [15]. Among these enzymes, MMP-9 exhibited the greatest upregulation, consistent with its capacity to degrade fibrillar collagens and elastin. Excessive MMP-9 activity is recognized to drive matrix disorganization and has been implicated in early atherogenesis and plaque instability [16]. The concurrent reduction in TIMP-1 amplifies this effect, producing an enzymatic milieu that facilitates valve leaflet remodeling and may accelerate the transition from a thickened, non-calcified valve to a calcified, stenotic lesion. Similar increases in circulating MMP-9 and MMP-9/TIMP-1 ratios have been associated with atherosclerotic plaque instability and adverse cardiovascular events [16].

From a metabolic standpoint, obesity was highly prevalent among patients with AS, nearly half of whom had a BMI ≥30 kg/m^2^, including several with morbid obesity. Obesity is known to exacerbate CAVD through chronic low-grade inflammation, metabolic dysregulation, and hemodynamic overload, all contributing to valvular and vascular remodeling [4, 17]. In our study, systolic blood pressure was significantly higher in AS than in NAV, consistent with increased left ventricular afterload secondary to valvular narrowing. Likewise, LDL-cholesterol levels were particularly elevated in the AVSc group, consistent with prior evidence that LDL accumulation and oxidation initiate endothelial dysfunction, immune cell recruitment, and cytokine release–hallmarks of both atherogenesis and early valvular sclerosis [18].

These observations parallel the pathobiological continuum linking AVSc and atherosclerosis–both characterized by lipid infiltration, immune activation, cytokine production, and MMP-mediated ECM degradation preceding calcification. Previous studies have shown that MMP activity promotes activation of the nuclear factor kappa-light-chain-enhancer of activated B cells (NF-κB) pathway and induces proinflammatory cytokines such as IL-6, IL-8, and monocyte chemoattractant protein-1 [6, 19]. Our results extend this paradigm by demonstrating that a distinct inflammatory and proteolytic signature is already established at the sclerosis stage, before overt hemodynamic obstruction occurs.

From a clinical perspective, these findings may have important implications. The coexistence of systemic inflammation and proteolytic activation in AVSc suggests that early therapeutic modulation may delay or prevent progression toward stenosis. Statins, for instance, possess anti-inflammatory and anti-MMP properties beyond lipid lowering [20, 21]. Although large clinical trials in established AS have been largely negative, their efficacy in earlier disease stages such as AVSc deserves renewed investigation [22, 23]. Likewise, experimental strategies targeting MMP activity (e.g., doxycycline) or inflammatory cytokines (e.g., TNF antagonists) could also stabilize the valvular matrix, though these approaches remain largely theoretical in valvular disease [16, 24]. Recognizing AVSc as an inflammatory phenotype rather than an incidental echocardiographic finding emphasizes the need for preventive interventions before irreversible calcification ensues. Moreover, AVSc has been linked to increased risk of coronary artery disease and acute coronary syndromes, reinforcing its role as a marker of systemic atherosclerotic burden [25].

This study has limitations. Its cross-sectional design precludes causal inference or assessment of progression from sclerosis to stenosis. Patients with AS were older and had more comorbidities, reflecting real-world demographics but complicating direct comparisons. Additionally, we quantified circulating biomarkers, which may not fully represent local valvular processes. Future studies integrating serum and tissue analyses could clarify whether systemic alterations mirror local molecular events. Finally, longitudinal studies are warranted to determine whether the biomarker profile identified here predicts progression to clinically significant stenosis.

In conclusion, this study provides evidence that AVSc may constitute an active inflammatory and proteolytic phase in the early continuum of CAVD. The combination of elevated TNF, increased MMP-9, and reduced TIMP-1 defines a biological signature of valvular inflammation and matrix remodeling that parallels early atherogenic mechanisms. Recognizing this stage as an active disease process rather than a benign, age-related finding opens new avenues for preventive and targeted strategies aimed at halting disease progression before irreversible calcification develops.

## Data Availability

The raw data supporting the conclusions of this article will be made available by the authors, without undue reservation.
